# Supplementary material for: Ethanol-Mediated Stress Promotes Autophagic Survival and Aggressiveness of Colon Cancer Cells via Activation of Nrf2/HO-1 Pathway
Source: Cancers (Basel). 2019 Apr 10;11(4):505. doi: 10.3390/cancers11040505 (PMC6521343; doi:10.3390/cancers11040505)
Supplement: Supplementary file 1 [file cancers-11-00505-s001.pdf]

Supplementary Materials:

Ethanol-Mediated Stress Promotes Autophagic Survival and Aggressiveness of Colon Cancer Cells Via Activation of Nrf2/HO-1 Pathway

Cesare Cernigliaro, Antonella D'Anneo, Daniela Carlisi, Michela Giuliano, Antonella Marino Gammazza, Rosario Barone, Lucia Longhitano, Francesco Cappello, Sonia Emanuele, Alfio Distefano, Claudia Campanella, Giuseppe Calvaruso, Marianna Lauricella

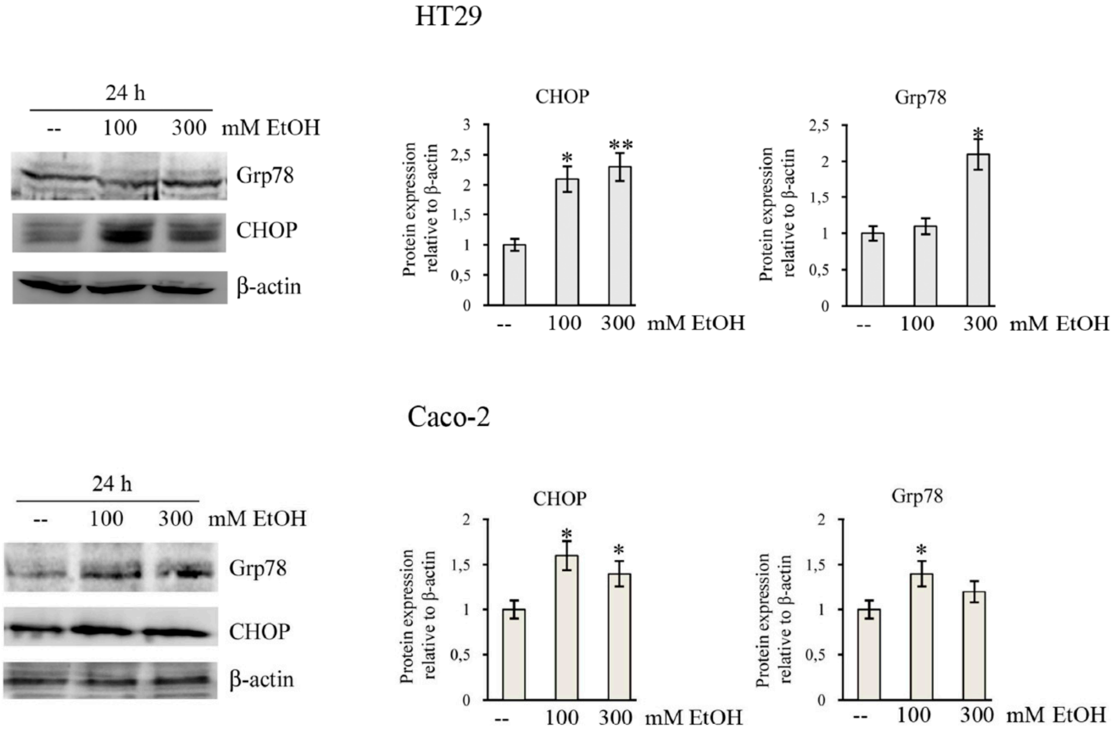

Figure S1. Ethanol induces ER stress in HT29 and Caco-2 colon cancer cells. Western blotting analysis of HOP and Grp78 in HT29 and Caco-2 cells treated for 24 h with 100 or 300 mM EtOH. The correct protein loading was ascertained by immunoblotting for β-actin. Representative blots of three independent experiments and densitometric analysis are shown. (*) $p < 0.05$ and (**) $p < 0.01$ compared to the untreated sample.

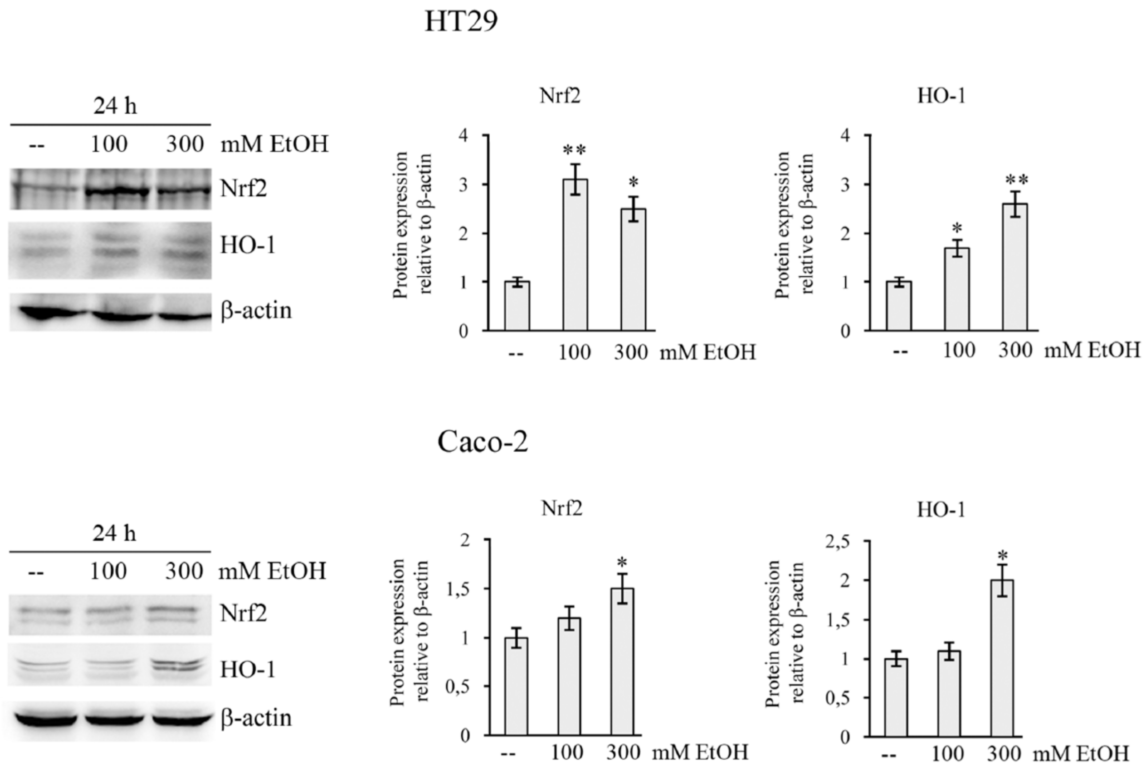

Figure S2. Effects of ethanol on Nrf2 and HO-1 in HT29 and Caco-2 colon cancer cells. Western blotting analysis of Nrf2 and HO-1 in HT29 and Caco-2 cells treated for 24 h with 100 or 300 mM EtOH. The correct protein loading was ascertained by immunoblotting for β-actin. Representative blots of three independent experiments and densitometric analysis are shown. (*) $p < 0.05$ and (**) $p < 0.01$ compared to the untreated sample.

© 2019 by the authors. Licensee MDPI, Basel, Switzerland. This article is an open access article distributed under the terms and conditions of the Creative Commons Attribution (CC BY) license (http://creativecommons.org/licenses/by/4.0/).
